# A ubiquitin switch controls autocatalytic inactivation of the DNA–protein crosslink repair protease SPRTN

**DOI:** 10.1093/nar/gkaa1224

**Published:** 2020-12-22

**Authors:** Shubo Zhao, Anja Kieser, Hao-Yi Li, Hannah K Reinking, Pedro Weickert, Simon Euteneuer, Denitsa Yaneva, Aleida C Acampora, Maximilian J Götz, Regina Feederle, Julian Stingele

**Affiliations:** Department of Biochemistry, Ludwig-Maximilians-University, 81377 Munich, Germany; Gene Center, Ludwig-Maximilians-University, 81377 Munich, Germany; Department of Biochemistry, Ludwig-Maximilians-University, 81377 Munich, Germany; Gene Center, Ludwig-Maximilians-University, 81377 Munich, Germany; Department of Biochemistry, Ludwig-Maximilians-University, 81377 Munich, Germany; Gene Center, Ludwig-Maximilians-University, 81377 Munich, Germany; Department of Biochemistry, Ludwig-Maximilians-University, 81377 Munich, Germany; Gene Center, Ludwig-Maximilians-University, 81377 Munich, Germany; Department of Biochemistry, Ludwig-Maximilians-University, 81377 Munich, Germany; Gene Center, Ludwig-Maximilians-University, 81377 Munich, Germany; Department of Biochemistry, Ludwig-Maximilians-University, 81377 Munich, Germany; Gene Center, Ludwig-Maximilians-University, 81377 Munich, Germany; Department of Biochemistry, Ludwig-Maximilians-University, 81377 Munich, Germany; Gene Center, Ludwig-Maximilians-University, 81377 Munich, Germany; Department of Biochemistry, Ludwig-Maximilians-University, 81377 Munich, Germany; Gene Center, Ludwig-Maximilians-University, 81377 Munich, Germany; Department of Biochemistry, Ludwig-Maximilians-University, 81377 Munich, Germany; Gene Center, Ludwig-Maximilians-University, 81377 Munich, Germany; Institute for Diabetes and Obesity, Monoclonal Antibody Core Facility, Helmholtz Zentrum München, 85764 Neuherberg, Germany; Department of Biochemistry, Ludwig-Maximilians-University, 81377 Munich, Germany; Gene Center, Ludwig-Maximilians-University, 81377 Munich, Germany

## Abstract

Repair of covalent DNA–protein crosslinks (DPCs) by the metalloprotease SPRTN prevents genome instability, premature aging and carcinogenesis. SPRTN is specifically activated by DNA structures containing single- and double-stranded features, but degrades the protein components of DPCs promiscuously and independent of amino acid sequence. This lack of specificity is useful to target diverse protein adducts, however, it requires tight control in return, in order to prohibit uncontrolled proteolysis of chromatin proteins. Here, we discover the components and principles of a ubiquitin switch, which negatively regulates SPRTN. We demonstrate that monoubiquitylation is induced in an E3 ligase-independent manner and, in contrast to previous assumptions, does not control chromatin access of the enzyme. Data obtained in cells and *in vitro* reveal that monoubiquitylation induces inactivation of the enzyme by triggering autocatalytic cleavage *in trans* while also priming SPRTN for proteasomal degradation *in cis*. Finally, we show that the deubiquitylating enzyme USP7 antagonizes this negative control of SPRTN in the presence of DPCs.

## INTRODUCTION

Covalent DNA–protein crosslinks (DPCs) are particularly dangerous DNA lesions, which interfere with all basic chromatin transactions including transcription and replication (1–3). Endogenous DPCs are not only caused by toxic metabolites such as reactive aldehydes but also by entrapment of covalent reaction intermediates of enzymes such as topoisomerases (4). Moreover, crosslinking can be induced by exogenous agents such as ionizing radiation as well as by various widely-used chemotherapeutics (5,6). The protein component of DPCs is targeted for repair by proteases of the Wss1/SPRTN family (7–14). The human protease SPRTN is essential for viability in mammalian cells, which highlights the scale of the threat posed by endogenous DPCs (15). Moreover, germline mutations in *SPRTN*, which result in the deletion of the enzyme's C-terminal tail, are causative for Ruijs-Aalfs syndrome (RJALS) (16,17). RJALS is characterized by premature aging and early-onset hepatocellular carcinomas with similar phenotypes being observed in hypomorphic *Sprtn* mutant mice (16–18). SPRTN is a DNA-dependent metalloprotease, which is activated by DNA structures containing single- (ss) and double-stranded (ds) features, such as ss-/dsDNA junctions or frayed dsDNA ends (19). However, SPRTN’s proteolytic activity is highly promiscuous (8,9,11). The lack of preference for certain amino acid sequences is required to target diverse DPCs, but it demands tight control in return. A substantial fraction of SPRTN (30-50%) is constitutively monoubiquitylated (20–22). The modification is strongly reduced in SPRTN variants with amino acid replacements in the enzyme's C-terminal ubiquitin-binding zinc finger (UBZ) (20–22). Attempts to identify the site of monoubiquitylation by mass spectrometry revealed four potentially-modified lysine (K) residues, but SPRTN variants with collective lysine-to-arginine (KR) substitutions retained the modification (8). It has been proposed that monoubiquitylation regulates chromatin access of the enzyme because the recruitment of SPRTN to chromatin upon DPC induction is accompanied by rapid deubiquitylation (8). However, testing this model directly, has not been possible because the involved deubiquitylating enzyme (DUB) is unknown. Accordingly, the mechanistic principles of SPRTN’s regulation by monoubiquitylation remain unclear.

Here, we identify the DUB USP7 as the factor responsible for deubiquitylating SPRTN when cells are challenged by DPCs. Moreover, we reveal that monoubiquitylation induces direct inactivation of SPRTN rather than regulating chromatin recruitment of the enzyme. The modification triggers autocleavage of the protease while also enhancing proteasomal degradation by priming polyubiquitylation. Finally, *in vitro* experiments suggest that the constitutive monoubiquitylation occurs in a highly promiscuous E3 ligase-independent manner. Taken together, we unravel the principles and components of a regulatory switch, which allows safe operation of a potentially dangerous enzymatic activity in human cells.

## MATERIALS AND METHODS

### Antibodies and inhibitors

Anti-Strep (ab76949) and anti-Histone H3 (ab10799) antibodies were purchased from Abcam; anti-Tubulin (T6074), anti-Flag (F3165) and anti-Rat IgG (A9037) antibodies were purchased from Sigma; anti-GAPDH (2118) antibody was purchased from Cell Signaling; anti-USP7 (sc-137008) and anti-Histone H1 (sc-377468) were purchased from Santa Cruz; Goat Anti-Mouse Immunoglobulins/HRP (P0447), Swine Anti-Rabbit Immunoglobulins/HRP (P0399) antibodies were purchased from Dako and anti-GFP (PABG1, used for detection of YFP) was purchased from Chromotek. Rat monoclonal anti-human SPRTN antibody (6F2) was generated by immunization of Wistar rats with purified GST-tagged SPRTN-ΔC, which was expressed in insect cells as described previously (8). Hybridoma supernatants were screened by ELISA for binding to purified untagged SPRTN protein and further validated by western blot analysis on HeLa cell lysates as well as recombinant protein. Clone SPRT 6F2 (IgG2a) was subcloned twice by limited dilution to obtain a stable monoclonal hybridoma cell line. For inhibition of the ubiquitin-activating enzyme E1 MLN7243 (TAK-243) was purchased from Chemietek and used at 1 μM final concentration (23). For inhibition of proteasomal degradation MG132 was purchased from Sigma (M7449) and used at 5 μM final concentration. For inhibition of protein synthesis cycloheximide was purchased from Sigma (C4859) and used at 100 μg/ml final concentration.

### Cell lines

HCT116 wild-type (WT), HCT116 *USP7* KO and HAP1 WT, *VCPIP1* KO, *USP11* KO cells were purchased from Horizon Discovery. HeLa T-REx Flp-In, 293 T-REx Flp-In and DLD1 cells were provided by Cell Services, The Francis Crick Institute. HeLa T-REx Flp-In cells stably expressing YFP-SPRTN-Strep-tag variants were generated using the Flp-In system (Thermo Fisher) according to manufacturer's instructions.

### Transient transfection

For transient transfections cells were grown to 70–90% confluency in 12-well plates. Plasmids (1 μg plasmid) and Lipofectamine 2000 reagent (Invitrogen, 1 μl/μg plasmid) were diluted in 50 μl Opti-MEM Medium each. Plasmid and Lipofectamine 2000 dilutions were mixed following a 5 min incubation. After an additional 15 min incubation, the transfection mix was added to the cells.

### siRNA transfection

Cells were grown to 20–30% confluency in 6-well plates. 3 μl siRNA (20 μM, ON TARGETplus SMARTpool, Horizon, USP7 (L-006097-00-0005), USP11 (L-006063-00-0005), VCPIP1 (L-019137-00-0005), Control (D-001810-10-05)) and 3 μl Lipofectamine RNAiMAX Transfection Reagent (Invitrogen) were each diluted in 100 μl Opti-MEM Medium. siRNA and Lipofectamine RNAiMAX Transfection Reagent dilutions were mixed following a 5 min incubation. After an additional 15 min incubation, the transfection mix was added to the cells. After 48 h, cells were reseeded into 60 mm dishes, followed by chromatin fractionation the following day.

### Generation of USP7 knock-out cells

USP7 gRNA1 (GGTCTGTCTGGATAAAAGCG) and gRNA2 (GAGTGATGGACACAACACCG) were inserted into Lenti-multi-CRISPR plasmid (Addgene #85402; RRID: Addgene_85402) as described previously (24). The resulting plasmid was then transiently transfected into HAP1 or DLD1 cells using Lipofectamine 2000 (Invitrogen) to generate USP7 knock-out cells. One day after transfection, cells were selected by Puromycin for 48 h. Selected cells were then reseeded in 96-well plates (0.5 cell/well) to generate single clones. Single clones were then screened using western blotting with anti-USP7 antibody.

### Purification of partially ubiquitylated YFP-SPRTN-EQ-Strep

293 T-REx Flp-In cells expressing YFP-SPRTN-EQ-Strep were grown in two 245 × 245 mm dishes to 50% confluency before overnight induction of protein expression by addition of 1 μg/ml doxycycline. Cells were harvested by scraping, washed twice in PBS and stored at −80°C. For purification, cells were thawed and resuspended in 10 ml lysis buffer (50 mM HEPES/NaOH pH 7.2, 1 M NaCl, 10% glycerol, 1% IGEPAL CA-630, cOmplete EDTA free protease inhibitors, 0.04 mg/ml PefaBloc SC and 20 mM iodoacetamide). Following sonication and Benzonase (4 U/ml) digestion for 30 min at 4°C, lysates were cleared by centrifugation (23 500 g, 45 min, 4°C). 60 μl MagStrep type3 XT beads (5% (v/v) suspension) were incubated with the supernatant for 1 h at 4°C prior to three wash steps with wash buffer (50 mM HEPES/NaOH pH 7.2, 0.5 M NaCl, 10% glycerol). Finally, purified YFP-SPRTN-EQ-Strep was eluted in 3 × 80 μl elution buffer (50 mM HEPES/NaOH pH 7.2, 0.5 M NaCl, 10% glycerol, 50 mM biotin).

### DUB screen

71 cDNAs encoding human DUBs (hORFeome v8.1 Deubiquitinating Enzymes collection + seven additional ORFeome clones: CloneIds: 100011387, 100010734, 100002718, 100066416, 100070362, 100068239) were sub-cloned into pDEST17 using Gateway LR Clonase II (Invitrogen). Plasmids were then transformed into *Escherichia coli* BL21 (DE3) cells for protein expression in 50 ml cultures. Cell pellets were resuspended in BugBuster reagent (Merck Millipore, 5 ml/g). Benzonase (25 U/ml) and DTT (5 mM) were added prior to an incubation of 20 min at room temperature. Lysates were then cleared by centrifugation (16 000 g, 20 min, 4°C). Lysates were mixed in pools of three prior to assessing their ability of deubiquitylating YFP-SPRTN-EQ-Strep. To this end, purified partially ubiquitylated YFP-SPRTN-EQ-Strep was incubated with lysate pools for 30 min at 25°C. Lysates of non-transformed BL21 cells served as negative control, the unspecific deubiquitylating activity of the catalytic domain of USP2 (USP2^cd^, BostonBiochem, E-504) as positive control. Deubiquitylation was then assessed using SDS-PAGE followed by western blotting using anti-Strep antibody.

### DUB activity assays

Candidate DUBs were partially purified using a standard Ni-NTA-purification strategy and then tested for their activity either using a ubiquitin–rhodamine cleavage assay kit (BostonBiochem) following the manufacture's instructions or by incubation with partially ubiquitylated YFP-SPRTN-EQ-Strep for 30 min at 25°C followed by SDS-PAGE and western blotting using anti-Strep antibody.

### Expression and purification of recombinant proteins

SPRTN codon-optimized for bacterial expression (using GeneOptimizer) was expressed from a pNIC-Strep-ZB-SPRTN plasmid as previously described (19). SPRTN-Ub^LF^ was generated using Gibson assembly cloning. Flag-SPRTN was generated using insertional mutagenesis. For protein expression plasmids were transformed into BL21(DE3) *E. coli* cells and grown at 37°C in Terrific broth (TB) medium until they reached OD 0.7. Protein expression was induced by addition of 0.5 mM IPTG over night at 18°C. Next, cells were harvested, resuspended in buffer A (50 mM HEPES/KOH pH 7.2, 500 mM KCl, 1 mM MgCl_2_, 10% glycerol, 0.1% IGEPAL, 0.04 mg/ml Pefabloc SC, cOmplete EDTA-free protease inhibitors, 1 mM Tris(2-carboxyethyl)phosphine hydrochloride (TCEP), pH 7.2) and lysed by sonication. All steps were carried out at 4°C. Cell lysate was incubated with benzonase (45 U/ml lysate) for 30 min on ice prior to the removal of cell debris by centrifugation at 18 000 g for 30 min. Cleared supernatant was applied to Strep-Tactin^®^XT Superflow^®^ high capacity cartridges, washed with 3 column volumes (CV) of buffer A and 4 CV of buffer B (50 mM HEPES/KOH pH 7.2, 500 mM KCl, 10% Glycerol, 1 mM TCEP, pH 7.2). Proteins were eluted in 6 CV buffer C (50 mM HEPES/KOH pH 7.2, 500 mM KCl, 10% glycerol, 1 mM TCEP and 50 mM biotin, pH 7.2). Eluted proteins were further applied to HiTrap Heparin HP affinity columns and washed with 3 CV buffer B before eluting in buffer D (50 mM HEPES/KOH pH 7.2, 1 M KCl, 10% glycerol, 1 mM TCEP, pH 7.2). Eluted fractions containing recombinant SPRTN protein were desalted against buffer B using PD-10 desalting columns. The affinity tag was cleaved off over night at 4°C by the addition of His-tagged TEV protease with 1:10 mass ratio. Cleaved recombinant SPRTN protein was further purified by size exclusion chromatography using a HiLoad 16/600 Superdex 200 pg column equilibrated in buffer E (50 mM HEPES/KOH pH 7.2, 500 mM KCl, 10% Glycerol, 0.5 mM TCEP, pH 7.2). Eluted proteins were concentrated with 10 kDa cutoff Amicon Ultra centrifugal filters before snap-freezing in liquid nitrogen and storing at −80°C.

UBE2D3 was sub-cloned into pDEST17 using Gateway LR Clonase II (Invitrogen). For protein expression plasmids were transformed into BL21(DE3) *E. coli* cells and grown at 37°C in Terrific broth (TB) medium until they reached OD 0.7. Protein expression was induced by addition of 0.5 mM IPTG for 3 h at 37°C. Next, cells were harvested, resuspended in buffer A (50 mM NaH_2_PO_4_ pH 8, 150 mM NaCl, 10 mM Imidazol and 0.5 mM TCEP), with the addition of 0.04 mg/ml Pefabloc SC and cOmplete EDTA-free protease inhibitors. The cells were then lysed by sonication. All steps were carried out at 4°C. Cell lysate was incubated with benzonase (45 U/ml lysate) for 30 min at 4°C prior to the removal of cell debris by centrifugation at 16 000 g for 30 min. Cleared supernatant was applied twice to Ni-NTA beads equilibrated in buffer A, washed with 5 CV of buffer A and 7 CV of buffer B (50 mM NaH_2_PO_4_ pH 8, 500 mM NaCl, 20 mM Imidazol, 0.5 mM TCEP). Proteins were eluted in 5 CV buffer C (50 mM NaH_2_PO_4_ pH 8, 500 mM NaCl, 250 mM Imidazol, 0.5 mM TCEP). Eluted fractions containing recombinant His-UBE2D3 protein were desalted against buffer D (20 mM Tris pH 7.5, 150 mM NaCl, 10% glycerol, 2 mM TCEP) using PD-10 desalting columns. The recombinant protein was further purified by size exclusion chromatography using a HiLoad 16/600 Superdex 200 pg column equilibrated in buffer D. Eluted proteins were concentrated with 3 kDa cutoff Amicon Ultra centrifugal filters before snap-freezing in liquid nitrogen and storing at −80°C.

### *In vitro* ubiquitylation assay

The E2 screen was conducted using the E2 screening kit (UBPBio) according to the manufacturer's instructions. In brief, human E2 ubiquitin conjugating enzymes (2 μM) were incubated together with catalytically inactive SPRTN-E112Q (EQ) (2 μM), E1 ubiquitin activating enzyme (100 nM), ubiquitin (R&D Systems, U-100H, 50 μM) or no lysines N-Terminal Biotin ubiquitin (R&D Systems, UB-NOK-050, 50 μM), DNA (11.1 nM ФX174 virion) and ATP (2 mM) for 1.5 h at 30°C. All other *in vitro* ubiquitylation assays contained the indicated SPRTN variants, E1 ubiquitin activating enzyme (300 nM), ubiquitin (50 μM), ATP (2 mM) and purified His-tagged UBE2D3 (concentrations as indicated in figure legends) and were incubated for 1.5 h at 30°C. The catalytic domain of USP2 (USP2^cd^) was purchased from BostonBiochem and was included as indicated. Ubiquitylation reactions were stopped by addition of 4× LDS sample buffer supplemented with β-mercaptoethanol and boiling at 95°C for 10 min and then subjected to SDS-PAGE followed by staining with InstantBlue Coomassie protein stain. Contrast of scanned images was adjusted globally using Adobe Photoshop software

### *In vitro* protease assays

Reactions were performed in 20 μl containing the indicated SPRTN variants (500 nM), histone H1 (500 nM, NEB) as indicated and either ФX174 Virion ssDNA or RFI dsDNA (11.1 nM, NEB). The reaction buffer comprised 19.5 mM HEPES/KOH pH 7.2, 2.9% glycerol, 5 mM TCEP and either 80 mM or 150 mM KCl. Reactions were incubated at 25°C for 1 h and stopped by the addition of 4x LDS sample buffer supplemented with β-mercaptoethanol. Samples were boiled at 95°C for 10 min, resolved on 4–12% Bis–Tris NuPAGE gradient gels and stained using InstantBlue Coomassie protein stain or analysed by western blotting using anti-SPRTN, anti-Flag and anti-H1 antibodies. The intensity of western blots and scanned gels was adjusted globally using Adobe Photoshop. Cleavage reactions were quantified by dividing the amount of cleaved protein by the total amount of protein (cleaved and uncleaved) as determined by analysis of western blot results using ImageJ.

### Protein-oligonucleotide conjugate cleavage assay

Protein G was conjugated to the 5′-terminal, 3′-terminal or an internal base of a 30mer oligonucleotide (5′-Cy5-ACCAGTGCCTTGCTAGGACATCTTTGCCCA-3′) as described previously (19). Double-stranded DPCs were generated by annealing a complementary reverse oligonucleotide. Annealing was carried out immediately prior to cleavage reactions by mixing conjugates and reverse oligonucleotides at a ratio of 1:1.2 in annealing buffer (25 mM HEPES/KOH pH 7.2, 50 mM KCl, 5% Glycerol, and 0.2 mg/ml BSA) followed by an incubation for 2 min at 37°C and a subsequent decrease in temperature of 1°C/min until 25°C were reached. Cleavage reactions with model DPCs were performed in a reaction volume of 10 μl containing 6.25 nM SPRTN and 25 nM DPC in a final reaction buffer of 17.5 mM HEPES/KOH pH 7.2, 80 mM KCl, 3.5% glycerol, 5 mM TCEP and 0.1 mg/ml BSA. Reactions were incubated for 2 h at 25°C. 2 μl of 6× Orange G loading dye was added before reactions were resolved on 20% TBE gels using 1× TBE as running buffer at 4°C. Gels were photographed using a BioRad Chemidoc MP system using filter settings for Cy5 fluorescence. The intensity of scanned gels was adjusted globally using ImageJ, which was also used to quantify cleavage by dividing the amount of cleaved conjugate by the total amount of conjugate (cleaved and uncleaved) and subtraction of background signal (determined from lanes without SPRTN).

### Co-immuno-precipitation

To test binding between USP7/VCPIP1/USP11 and SPRTN, HeLa-T-REx Flp-In cells stably expressing YFP-SPRTN-Strep variants were seeded in 60 mm tissue culture plates, grown to 50% confluency and then transiently transfected with pcDNA5-FRT/TO plasmids encoding Flag/Flag-USP7/VCPIP1/USP11 variants using Lipofectamine 2000 (Invitrogen) according to the manufacturer's instructions. Sixteen hours after transfection and concurrent induction of protein expression by doxycycline (1 μg/ml), cells were washed once with ice-cold PBS and harvested by scraping in lysis buffer (20 mM Tris/HCl pH 7.5, 137 mM NaCl, 1% IGEPAL CA-630, 2 mM EDTA, 2 mM MgCl_2_, 4 U/ml Benzonase, cOmplete EDTA free protease inhibitors, 0.04 mg/ml PefaBloc SC and 20 mM iodoacetamide). Lysates were incubated for 30 min on ice, before centrifugation at 16 000 g for 30 min. Supernatants were then used for immuno-precipitation using 5 μl magnetic anti-Flag M2 beads (Sigma) at 4°C for 1 h. The beads were then washed three times with wash buffer (10 mM Tris/HCl pH 7.5, 150 mM NaCl, 0.5 mM EDTA and 0.5% IGEPAL CA-630). Finally, samples were resuspended in 100 μl 1× LDS sample buffer, analysed by SDS-PAGE and western blotting with anti-Flag, anti-GAPDH and anti-Strep antibodies.

### Cellular autocleavage assay

pcDNA5-FRT/TO plasmids encoding YFP-SPRTN-Strep or YFP-SPRTN-Ub^LF^ variants (1 μg) and Flag/Flag-USP7^WT/C223S^ (3 μg) were transiently transfected using Lipofectamine 2000 (Invitrogen) according to the manufacturer's instructions. Protein expression was induced by overnight (16 h) incubation with doxycycline (final concentration 1 μg/ml). Cells were lysed on ice in 1 ml lysis buffer (50 mM HEPES pH 7.5, 1 M NaCl, 10% glycerol, 1% IGEPAL CA-630, 2 mM MgCl_2_, 20 mM iodoacetamide, 0.04 mg/ml PefaBloc SC and cOmplete EDTA-free protease inhibitors). After addition of benzonase (4 U/ml), lysates were incubated for 30 min on ice. Lysates were cleared by centrifugation (16 000 g, 30 min) at 4°C and applied to 15 μl GFP-trap Magnetic Agarose (Chromotek) and processed according to manufacturer's instructions. Finally, samples were resuspended in 65 μl 1× LDS sample buffer, subjected to analysis by SDS-PAGE and western blotting with anti-GFP antibody (PABG1, Chromotek).

### Chromatin fractionation

Cells in the mid-exponential phase of growth were collected by scraping in ice-cold 1X PBS. Cells were then equally split and either directly resuspended in 1X LDS buffer or incubated for 10 min in ice-cold CSK buffer (10 mM PIPES, 100 mM NaCl, 1.5 mM MgCl_2_, 5 mM EDTA, 300 mM sucrose and 0.5% Triton X-100, 0.04 mg/ml PefaBloc SC and cOmplete EDTA free protease inhibitors). Chromatin-bound proteins were then isolated by low speed centrifugation (3000 rpm, 5 min at 4°C).

### Cycloheximide chase

Cells were seeded in 12-well tissue culture plates, grown to 80% confluency and then treated with 5 μM MG132. After 2 h cells were treated with 100 μg/ml cycloheximide (Sigma) for the indicated amount of time. Finally, cells were lysed in 150 μl 1X LDS sample buffer, followed by SDS-PAGE and western blotting with the indicated antibodies.

### Formaldehyde sensitivity assay

Long term treatment: 10^4^ cells were seeded per well in 12-well plates and treated with the indicated formaldehyde concentration the next day. After 72 h, medium was replaced with alamarBlue cell viability reagent (36 μg/ml resazurin in PBS) and plates kept for an additional 1 h incubation at 37°C. Cell viability was then assessed by measuring fluorescence (560 nm excitation/590 nm emission). Short term treatment: 5 × 10^4^ cells were seeded per well in 12-well plates and treated with 1 mM formaldehyde concentration the next day for 2 h. After 48 h, medium was replaced with alamarBlue cell viability reagent (36 μg/ml resazurin in PBS) and plates kept for an additional 1 h incubation at 37°C. Cell viability was then assessed by measuring fluorescence (560 nm excitation/590 nm emission).

### Detection of formaldehyde-induced DNA–protein crosslinks

DPCs were induced by treating HAP1 WT or *USP7* KO cells with 75 μM formaldehyde for 2 h. DPCs were measured using a KCl/SDS precipitation assay as described before (25). Briefly, cells were washed twice with PBS and lysed in 400 μl denaturing lysis buffer (2% SDS, 20 mM Tris/HCl, pH 7.5), frozen in liquid nitrogen and stored at −80°C until further processing. Lysates were thawed at 55°C for 5 min with 1200 rpm shaking, followed by pipetting samples up and down 30 times. Cellular protein was then precipitated by adding 400 μl precipitation buffer (200 mM KCl, 20 mM Tris pH 7.5) and incubation on ice for 5 min. The precipitated protein was separated by full speed centrifugation at 4°C for 5 min. Next, 400 μl supernatant was saved and used for soluble DNA measurement. The pellet was resuspended in 400 μl precipitation buffer and resolved by shaking at 55°C for 5 min followed by cooling down on ice for 5 min and full speed centrifugation at 4°C for 5 min. After repeating the wash procedure 3 times, protein precipitate was resuspended in 400 μl Proteinase K buffer (200 mM KCl, 20 mM Tris pH 7.5. Proteinase K 0.2 mg/ml) and incubated at 55°C for 45 min. Finally, 10 μl BSA (50 mg/ml) was added to the solution followed by cooling down on ice for 5 min followed full speed centrifugation at 4°C for 5 min. Next, supernatant containing crosslinked DNA was collected. Total DNA and crosslinked DNA were treated with 0.2 mg/ml RNase A for 30 min at 37°C. DNA concentrations were determined using Qubit™ dsDNA HS Assay Kit (Thermo Fisher) according to the manufacturer's instructions. The amount of DPCs was calculated as the ratio between crosslinked DNA and total DNA (crosslinked plus soluble DNA).

### Complementation of *USP7* KO cells

DLD1 WT or *USP7* KO cells were seeded in six-well plates, grown to 50% confluency before transient transfection with pcDNA5-FRT/TO plasmids encoding YFP/YFP-USP7^WT/C223S^ (2 μg) using Lipofectamine 2000 (Invitrogen) according to the manufacturer's instructions. 16 h after transfection, cells were reseeded into 12-well plates (5 × 10^4^ cells per well) followed by treatment with 1 mM formaldehyde for 2 h the next day. After 48 h cell viability was determined using alamarBlue cell viability assay.

## RESULTS

### The monoubiquitylation of SPRTN’s C-terminal tail is promiscuous

**Figure 1.**
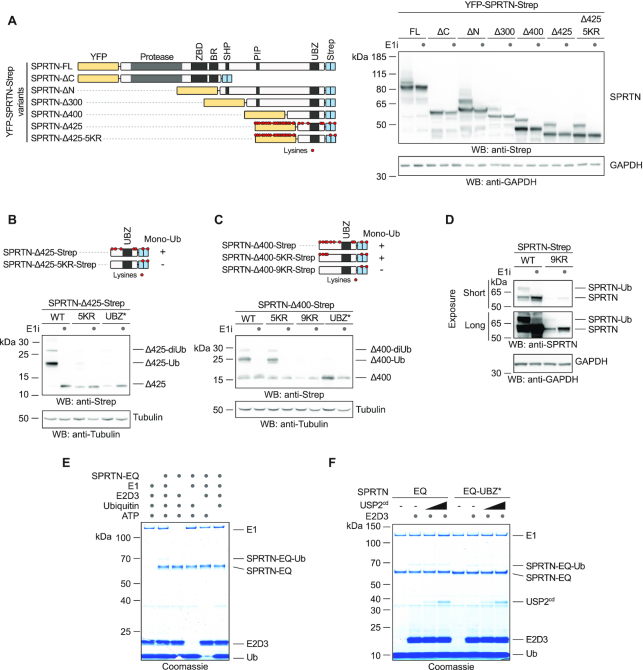
Promiscuous E3-independent monoubiquitylation of SPRTN’s C-terminal tail. (**A–D**) Monoubiquitylation status of truncated SPRTN variants. Plasmids encoding tagged full-length (FL) SPRTN or truncations (carrying the indicated lysine to arginine (KR) substitutions or the UBZ* variant, D473A) were transiently transfected in HeLa T-REx Flp-In cells. Expression of SPRTN was induced by addition of doxycycline for 6 h prior to cell lysis (including a co-treatment with a ubiquitin-activating enzyme E1 inhibitor (E1i) as indicated) and analysed by SDS-PAGE and western blotting. (**E**) *In vitro* ubiquitylation assays containing SPRTN-EQ (410 nM), UBE2D3 (4 μM), E1 ubiquitin activating enzyme (300 nM), ubiquitin (50 μM) and ATP (2 mM) as indicated were incubated for 1.5 h at 30°C. Reactions were stopped by addition of LDS sample buffer and subjected to SDS-PAGE followed by staining with InstantBlue Coomassie protein stain. (**F**) *In vitro* ubiquitylation assays containing SPRTN-EQ or SPRTN-EQ-UBZ* (410 nM), E1 ubiquitin activating enzyme (300 nM), ubiquitin (50 μM), ATP (2 mM), UBE2D3 as indicated (8 μM) and increasing amounts of the catalytic domain of USP2 (USP2^cd^) (0, 250 or 500 nM) were incubated for 1.5 h at 30°C. Reactions were stopped by addition of LDS sample buffer and subjected to SDS-PAGE followed by staining with InstantBlue Coomassie protein stain.

In order to understand the regulation of SPRTN by monoubiquitylation, we first attempted to map the modified lysine residue(s) using protein truncations. Ubiquitylation of full length SPRTN (N-terminally YFP-, C-terminally Strep-tagged) is readily observed upon transient transfection and is sensitive to inhibition of the ubiquitin-activating enzyme (E1) (Figure 1A). The truncated SPRTN variant found in RJALS patients (SPRTN-ΔC) is not ubiquitylated, while the isolated C-terminal tail (SPRTN-ΔN) is modified (Figure 1A). The modification can be further mapped to a small 7 kDa fragment, which contains the UBZ domain and five lysine residues (SPRTN-Δ425). Surprisingly, substitution of all five lysines (5KR) does not alter the level of modification (Figure 1A). The N-terminal YFP-tag and linker contain various lysines and we suspected that these residues might undergo modification as well. Indeed, deletion of the YFP-tag leads to a severe reduction in ubiquitylation of the SPRTN-Δ425-5KR fragment (Figure 1B). However, a slightly extended variant (SPRTN-Δ400) with the same lysine replacements (SPRTN-Δ400-5KR) remains ubiquitylated, unless all additional lysines are replaced as well (SPRTN-Δ400-9KR) (Figure 1C). Full-length SPRTN with the 9KR replacement is unstably expressed, but appears to remain monoubiquitylated (Figure 1D). Notably, a SPRTN-Δ400-8KR fragment with only one remaining lysine residue displays multiple modifications, which indicates that the monoubiquitylation can be further modified (Supplementary Figure S1A). We conclude that the monoubiquitylation of SPRTN can target various lysine residues (even those of the YFP-tag) and can be extended to a ubiquitin chain.

### E3-independent monoubiquitylation of SPRTN

Monoubiquitylation of proteins bearing ubiquitin-binding domains is frequently observed and has been proposed to occur in an E3-independent manner (26–28). Thus, we tested a collection of human recombinant ubiquitin-conjugating enzymes (E2s) for their ability to ubiquitylate catalytically-inactive SPRTN *in vitro*. Strikingly, ten out of twenty-nine E2s induce SPRTN monoubiquitylation in the absence of an E3 ubiquitin ligase (Figure 1E and Supplementary Figure S1B). *In vitro* ubiquitylation by the E2 UBE2D3 even triggers multi-monoubiquitylation of SPRTN, as indicated by multiple modifications with a ubiquitin variant containing no lysines and a biotinylated N-terminus (Supplementary Figure S1C). Notably, a SPRTN variant with an altered UBZ domain (UBZ*, D473A) undergoes modification *in vitro*, but its modification is sensitive to the addition of an unspecific deubiquitylation activity (USP2 catalytic domain) (Figure 1F). This could indicate that SPRTN-UBZ* variants lack monoubiquitylation in cells because a functional UBZ domain is important to shield the modification from cellular DUB activities. The fact that monoubiquitylation of SPRTN occurs in an E3-independent manner (although an involvement of E3 ligases in cells cannot be excluded) and the high level of modification in basal conditions argues that the generation of ubiquitylated SPRTN (SPRTN-Ub) is a constitutive process. In turn, this infers that cellular control of the modification must occur through deubiquitylation.

### An *in vitro* screen reveals that USP7 targets ubiquitylated SPRTN

**Figure 2.**
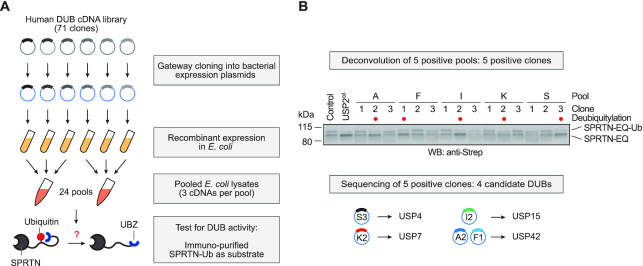
An *in vitro* screen identifies DUBs targeting ubiquitylated SPRTN. (**A**) Schematic depiction of the screening strategy employed to test seventy-one human deubiquitylating enzymes (DUBs) for their ability to deubiquitylate SPRTN. (**B**) Deconvolution of positive pools identified in Supplementary Figure S2A reveals four candidate DUBs. Lysates prepared from fifteen clones present in the five positive pools were incubated for 30 min at 25°C together with purified partially-monoubiquitylated YFP-SPRTN-EQ-Strep. Reactions were stopped by addition of LDS sample buffer and analysed by SDS-PAGE and western blotting using anti-Strep antibody. Lysates of BL21 cells served as negative control, the unspecific deubiquitylating activity of the catalytic domain of USP2 (USP2^cd^) as positive control.

**Figure 3.**
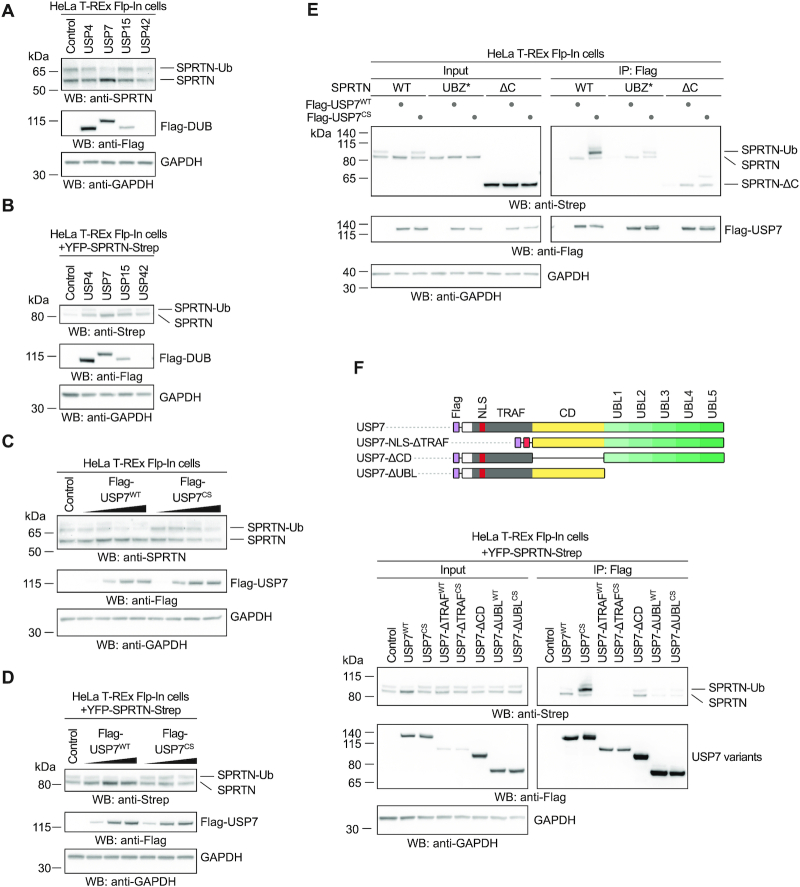
USP7 interacts with and targets ubiquitylated SPRTN in human cells. (**A**) Analysis of DUB overexpression-induced deubiquitylation of endogenous SPRTN in HeLa T-REx Flp-In cells. Indicated N-terminally Flag-tagged DUBs were transiently expressed for one day before cells were lysed and analysed by western blotting. (**B**) Analysis of DUB overexpression-induced deubiquitylation of doxycycline-inducible YFP-SPRTN-Strep stably expressed in HeLa T-REx Flp-In cells. Indicated N-terminally Flag-tagged DUBs were transiently expressed for one day before cells were lysed and analysed by western blotting. (**C**) Increasing amounts of N-terminally Flag-tagged USP7 (or the catalytically inactive CS variant) were transiently expressed in HeLa T-REx Flp-In cells for one day before cells were lysed and analysed by western blotting. (**D**) Increasing amounts of N-terminally Flag-tagged USP7 (or the catalytically inactive CS variant) were transiently expressed in HeLa T-REx Flp-In cells stably expressing doxycycline-inducible YFP-SPRTN-Strep for one day before cells were lysed and analysed by western blotting. (**E**) Plasmids encoding Flag-tagged full-length USP7 (WT or the catalytically inactive CS variant) were transiently transfected in HeLa T-REx Flp-In cells stably expressing the indicated doxycycline-inducible YFP-SPRTN-Strep variants. Binding was analysed by co-immunoprecipitation using anti-Flag beads followed by western blotting. (**F**) Schematic depiction of USP7’s domain structure and protein truncations used for co-immunoprecipitation analysis with SPRTN (upper panel). Plasmids encoding Flag-tagged full-length USP7 (WT or the catalytically inactive CS variant) or the respective truncations were transiently transfected in HeLa T-REx Flp-In cells stably expressing doxycycline-inducible YFP-SPRTN-Strep. Binding was analysed by co-immunoprecipitation using anti-Flag beads followed by western blotting (lower panel).

To identify the DUB responsible for deubiquitylating SPRTN, we designed an *in vitro* screen (Figure 2A). We subcloned an arrayed cDNA library containing sequences of seventy-one human DUBs into bacterial expression plasmids. Upon expression in *E. coli*, lysates were prepared and pooled in twenty-four sets of three. Deubiquitylation activity was assessed by incubating each pool with partially monoubiquitylated SPRTN-EQ purified from human cells. Addition of five out of twenty-four pools triggered SPRTN deubiquitylation (Supplementary Figure S2A). Each positive pool contained one lysate able to deubiquitylate SPRTN (Figure 2B). The respective plasmids were re-isolated and determined to encode four different DUBs: USP4, USP7, USP15 and USP42. All four candidates were re-expressed, partially purified and successfully re-tested for their ability to deubiquitylate SPRTN (Supplementary Figure S2B-C). To test whether these DUBs possess the ability to act on SPRTN-Ub in cells, we monitored SPRTN monoubiquitylation in cells upon overexpression of the respective candidates. Overexpression of USP7 but not of the other candidates leads to a loss of endogenous and exogenously-expressed SPRTN-Ub and a concurrent increase in unmodified SPRTN (Figure 3A-B). Of note, whether USP42 can deubiquitylate SPRTN in cells remains unclear, given that it was not expressed at significant levels. Importantly, overexpression of a catalytically inactive variant of the DUB (USP7^CS^, C223S) does not trigger deubiquitylation (Figure 3C-D). Consistently, USP7 binds to SPRTN in co-immunoprecipitation experiments (Figure 3E). Notably, catalytically inactive USP7 (USP7^CS^) binds preferentially to ubiquitylated SPRTN. Interestingly, monoubiquitylated species of SPRTN-UBZ* and SPRTN-ΔC co-immunoprecipitate with USP7^CS^, although this modification is not detectable in input samples (Figure 3E). This observation is in agreement with our finding that SPRTN-UBZ* can be monoubiquitylated *in vitro*, but then fails to protect the modification (Figure 1F). USP7 bears an N-terminal TRAF domain, which precedes the catalytic domain (CD) and five C-terminal ubiquitin-like domains (UBLs). Deletion of the CD or the UBLs abrogates preferential binding of USP7 to SPRTN-Ub indicating that these domains are important to provide specificity for modified SPRTN, but are not essential for the interaction per se (Figure 3F). A USP7 variant lacking the TRAF domain (USP7-ΔTRAF) is deficient in SPRTN binding. However, interpretation of this result is complicated by the fact that this truncation is expressed at low levels, which may indicate more general defects (Figure 3F). We conclude that USP7 interacts specifically with SPRTN-Ub and has the ability to deubiquitylate the protease *in vitro* and in cells.

### USP7 deubiquitylates SPRTN upon DPC induction

**Figure 4.**
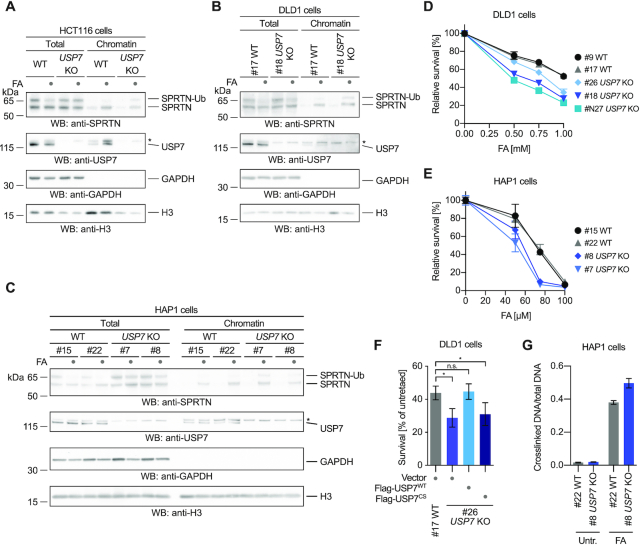
USP7 deubiquitylates SPRTN upon DPC induction. (**A**) HCT116 WT or *USP7* knock-out (KO) cells were treated with 2 mM formaldehyde (FA) for 2 h. Cells were either lysed directly in LDS sample buffer (total) or subjected to chromatin fractionation. Samples were then analyzed by SDS-PAGE followed by western blotting. Asterisks indicates a cross-reactive band. (**B**) Clonal DLD1 *USP7* KO cells and matched WT control cells were treated with 2 mM formaldehyde (FA) for 3 h. Cells were either lysed directly in LDS sample buffer (total) or subjected to chromatin fractionation. Samples were then analysed by SDS-PAGE followed by western blotting. Asterisks indicates a cross-reactive band. (**C**) Clonal HAP1 *USP7* KO cells and matched WT control cells were treated with 2 mM formaldehyde (FA) for 2 h. Cells were either lysed directly in LDS sample buffer (total) or subjected to chromatin fractionation. Samples were then analysed by SDS-PAGE followed by western blotting. Asterisks indicates a cross-reactive band. (**D**) Clonal DLD1 *USP7* KO cells and matched WT control cells were treated with the indicated formaldehyde concentrations for 2 h. After 48 h cell viability was determined using the alamarBlue cell viability assay. Values represent the mean ± SD of three technical replicates normalized to the mean of untreated controls of each cell line (**E**) Clonal HAP1 *USP7* KO cells and matched WT control cells were treated with the indicated formaldehyde concentrations for 72 h. Cell viability was then determined using the alamarBlue cell viability assay. Values represent the mean ± SD of three technical replicates normalized to the mean of untreated controls of each cell line. (**F**) YFP-tagged full-length USP7 (WT or the catalytically inactive CS variant) or the empty vector were transiently transfected in DLD1 *USP7* KO cells and matched WT control cells. Cells were treated with 1 mM formaldehyde for 2 h. After 48 h cell viability was determined using the alamarBlue cell viability assay. Values represent the mean ± SEM of four independent biological replicates normalized to the mean of untreated controls of each cell line. Significance was determined using a paired *t*-test (*P-value < 0.05). (**G**) Cellular DPCs were quantified in clonal HAP1 *USP7* KO cells and matched WT control cells treated with 75 μM formaldehyde for 2 h using a KCl/SDS precipitation assay. DPCs were measured as the ratio of crosslinked DNA compared to total DNA. Values represent the mean ± SD of three technical replicates.

Next, we tested whether USP7 is the DUB responsible for regulating SPRTN’s chromatin access by deubiquitylation upon DPC induction. Thus, we treated HCT116 WT and *USP7* knock-out (KO) cells for 2 h with formaldehyde before assessing recruitment of SPRTN by chromatin fractionation. Indeed, endogenous SPRTN fails to be deubiquitylated in the absence of USP7 upon formaldehyde exposure (Figure 4A). Unexpectedly however, the lack of deubiquitylation does not result in impaired recruitment. In *USP7* KO cells, also SPRTN-Ub is found on chromatin. These results indicate that deubiquitylation occurs downstream or in parallel to recruitment and is not preceding SPRTN’s relocalization to chromatin. To understand the contribution of USP7-mediated deubiquitylation to DPC repair, we generated DLD1 and HAP1 *USP7* KO cells because sensitivity of HCT116 *USP7* KO cells is difficult to assess due to their strong growth defect. HAP1 and DLD1 *USP7* KO cells show defective SPRTN deubiquitylation and hypersensitivity towards formaldehyde exposure (Figure 4B–E). Importantly, the formaldehyde sensitivity of DLD1 *USP7* KO cells is complemented by transient transfection of USP7^WT^ but not of USP7^CS^ (Figure 4F and Supplementary Figure S3A). Furthermore, HAP1 *USP7* KO cells accumulate higher levels of DPCs following a 2-h exposure to formaldehyde as determined using a KCl-SDS precipitation assays (Figure 4G).

Of note, while this study was under consideration, it was proposed that SPRTN is deubiquitylated by VCPIP1 (29). In addition, a recent preprint argued that USP11 is responsible for SPRTN deubiquitylation (30). Given our identification of USP7, we compared the contribution of all three enzymes to SPRTN deubiquitylation. Neither VCPIP1 nor USP11 induce SPRTN deubiquitylation when overexpressed, while USP7 does (Supplementary Figure S3B). VCPIP1 and USP11 interact weakly with SPRTN in co-immunoprecipitating experiments, but show no (VCPIP1) or only weak (USP11) preference for SPRTN-Ub (Supplementary Figure S3B). siRNA-mediated depletion of VCPIP1 or USP11 has no effect on SPRTN deubiquitylation in DLD1 cells, while depletion of USP7 does (Supplementary Figure S3C). Moreover, we obtained HAP1 *USP11* and *VCPIP1* KO cells which show sensitivity towards formaldehyde but no defects in SPRTN deubiquitylation (Supplementary Figure S3D-E).

To conclude, under the conditions tested here, USP7 but not VCPIP1 or USP11 has a prominent role in deubiquitylating SPRTN in cells. Finally, the formaldehyde sensitivity and DPC accumulation observed in *USP7* KO cells argue that deubiquitylation, although not involved in SPRTN’s chromatin recruitment, must have an important function in DPC repair. Therefore, we further explored the effects of monoubiquiytation on the SPRTN protease.

### Monoubiquitylation promotes degradation and autocleavage of SPRTN

**Figure 5.**
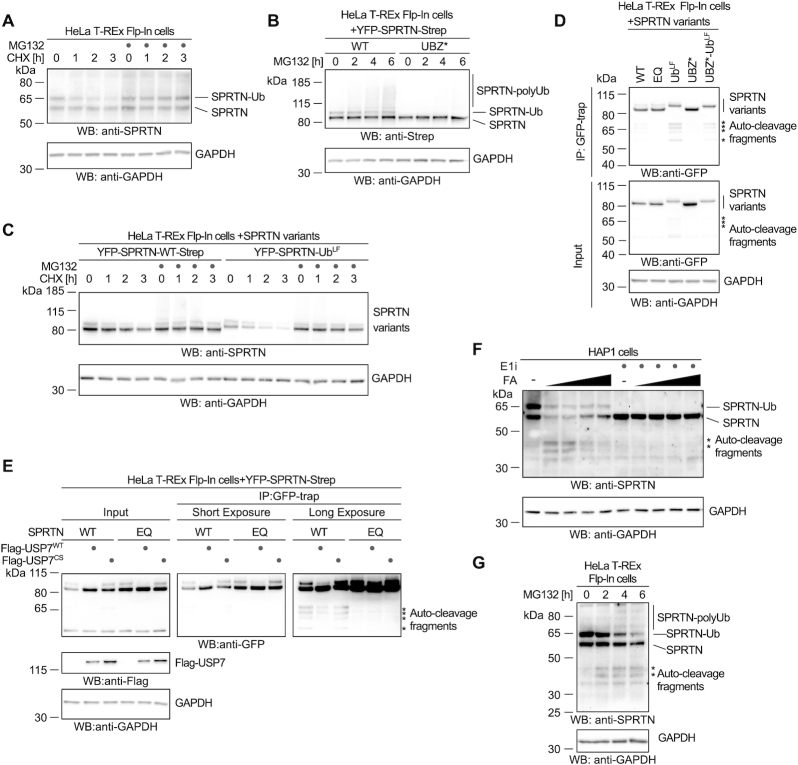
Monoubiquitylation promotes SPRTN degradation and autocleavage. (**A**) Stability of endogenous SPRTN was determined with a cycloheximide-chase experiment in HeLa-T-REx Flp-In cells. Cells were incubated with cycloheximide for the indicated amount of time (with or without a 2-h pre-treatment with the proteasome inhibitor MG132) prior to cell lysis and analysis by western blotting. (**B**) Polyubiquitylation of stably expressed doxycycline-inducible YFP-SPRTN-Strep or of YFP-SPRTN-UBZ*-Strep was determined in HeLa-T-REx Flp-In cells upon treatment with proteasome inhibitor MG132 for the indicated amount of time prior to cell lysis and analysis by western blotting. (**C**) Stability of stably expressed doxycycline-inducible YFP-SPRTN-Strep or a linear SPRTN-Ubiquitin fusion (YFP-SPRTN-Ub^LF^) was determined in HeLa-T-REx Flp-In cells using a cycloheximide-chase experiment. Cells were incubated in the presence of cycloheximide for the indicated amount of time (with or without a 2-h pre-treatment with the proteasome inhibitor MG132) prior to cell lysis and analysis by western blotting. (**D**) Indicated YFP-SPRTN-Strep or linear SPRTN-Ubiquitin fusion (YFP-SPRTN-Ub^LF^) variants were transiently transfected in HeLa-T-REx Flp-In cells. SPRTN autocleavage fragments were enriched on GFP-trap resins, followed by western blotting against the N-terminal YFP-tag. Western blotting of cell lysates against GAPDH serves as loading control. Asterisks indicate autocleavage fragments. (**E**) Indicated YFP-SPRTN-Strep variants were transiently transfected in HeLa-T-REx Flp-In cells in combination with Flag-tagged full-length USP7 (WT or the catalytically inactive CS variant) or the empty vector. SPRTN autocleavage fragments were enriched on GFP-trap resins, followed by western blotting against the N-terminal YFP-tag. Western blotting against GAPDH of cell lysates serves as loading control. Asterisks indicate autocleavage fragments. (**F**) HAP1 cells were treated with increasing amounts of formaldehyde (FA, 0.25, 0.5, 1 and 2 mM) for 2 h (either with or without a 2-h pre-treatment with ubiquitin-activating enzyme E1 inhibitor as indicated) prior to cell lysis and analysis by western blotting. Asterisks indicate autocleavage fragments. (**G**) HeLa-T-REx Flp-In cells were treated with proteasome inhibitor MG132 for the indicated amount of time prior to cell lysis and analysis by western blotting. Asterisks indicate autocleavage fragments.

Monoubiquitylation can lead to proteasomal degradation by priming polyubiquitylation (31,32). Thus, we assessed whether monoubiquitylation destabilizes SPRTN using cycloheximide-chase experiments. Indeed, endogenous SPRTN-Ub has a shorter half-life than non-ubiquitylated SPRTN, with degradation being blocked by proteasome inhibition (Figure 5A and Supplementary Figure S4A). Degradation is not affected by loss of USP7, which indicates that deubiquitylation is not involved in SPRTN protein stability under basal conditions (Supplementary Figure S4A). Proteasomal inhibition leads to accumulation of polyubiquitylated SPRTN species, which are strongly reduced in the SPRTN-UBZ* variant, which provides further support for monoubiquitylation inducing degradation by priming polyubiquitylation (Figure 5B and Supplementary Figure S4B). Furthermore, a linear fusion of SPRTN with ubiquitin (SPRTN-Ub^LF^, omitting the two C-terminal glycines), which has been previously suggested to mimic monoubiquitylation, destabilizes the entire SPRTN pool (Figure 5C) (21).

While conducting these experiments, we noted that cells expressing SPRTN-Ub^LF^ display various protein fragments, which are recognized by an antibody specific for the N-terminal YFP-tag (Figure 5D). These fragments become even more obvious when enriched on GFP-trap resins and correspond to the previously reported autocatalytic fragments seen in cells, which express WT SPRTN but are absent in cells expressing catalytically inactive SPRTN-EQ (Figure 5D) (8,9). These results raise the possibility that monoubiquitylation of SPRTN triggers enhanced autocleavage of the enzyme. In agreement, autocleavage of the non-ubiquitylated SPRTN-UBZ* variant is barely detectable, unless linearly-fused to ubiquitin (SPRTN-UBZ*-Ub^LF^) (Figure 5D). Furthermore, deubiquitylation of SPRTN induced by overexpression of USP7^WT^, but not of catalytically inactive USP7^CS^, leads to reduced formation of autocatalytic SPRTN fragments (Figure 5E). Autocleavage of endogenous SPRTN is induced by formaldehyde exposure and is more prominent at lower concentrations while deubiquitylation is observable at higher concentrations (Figure 5F) (8). Remarkably, if monoubiquitylation of endogenous SPRTN is blocked by pre-treating cells with ubiquitin E1 inhibitor, autocleavage is strongly reduced (Figure 5F). Interestingly, autocleavage of endogenous SPRTN also increases in cells, which have been treated with proteasome inhibitors (Figure 5G and Supplementary Figure S4C and D). This observation provides support for a model in which monoubiquitylated SPRTN is either degraded by the proteasome or undergoes autocleavage. In agreement, the short half-life of SPRTN-Ub is independent of the enzyme's catalytic activity and a SPRTN truncation (SPRTN^aa1-227^) corresponding to the shortest autocleaved fragment is not particularly unstable (Supplementary Figure S4E-F). Taken together, proteasomal degradation and autocleavage appear to be independent outcomes induced by monoubiquitylation.

**Figure 6.**
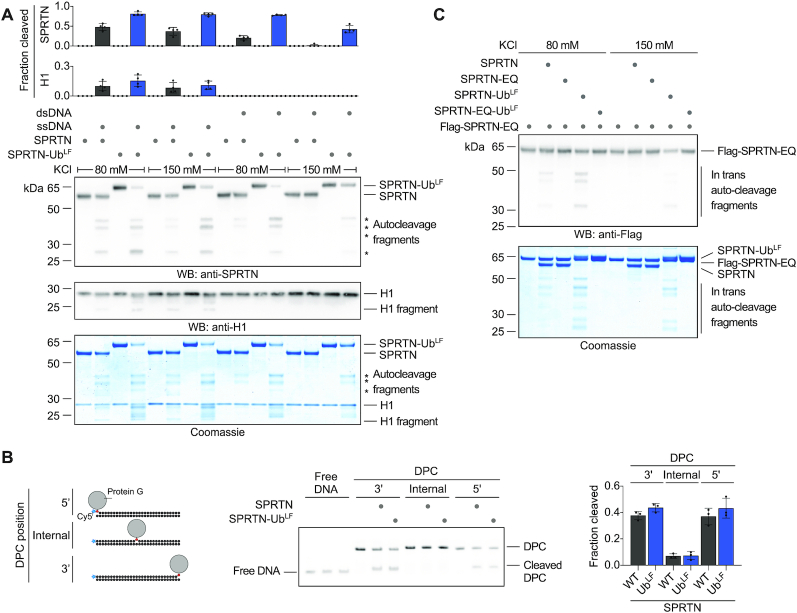
Monoubiquitylation promotes SPRTN autocleavage *in trans*. (**A**) Recombinant SPRTN or a linear SPRTN-Ubiquitin fusion (SPRTN-Ub^LF^) (500 nM) were incubated with histone H1 alone or in the presence of either single- (ss) Virion or double-stranded (ds) RFI ФX174 DNA (11.1 nM) for 60 min at 25°C. Salt concentrations were as indicated. Reactions were analysed by SDS-PAGE followed by western blotting and staining with InstantBlue Coomassie protein stain. Quantification of western blots of results of SPRTN and histone H1 cleavage: values represent the mean ± SD of four independent experiments. (**B**) Indicated model protein G-oligonucleotide conjugates (25nM) were incubated alone or in the presence of recombinant SPRTN (6.25 nM, WT or a linear SPRTN-Ubiquitin fusion (SPRTN-Ub^LF^)) for 2 h at 25°C prior to separation by native PAGE. Right panel, quantification of DPC cleavage: values represent the mean ± SD of three independent experiments. (**C**) Recombinant catalytically inactive Flag-SPRTN-EQ (500 nM) was incubated alone or in combination with active SPRTN (500 nM, WT or a linear SPRTN-Ubiquitin fusion (SPRTN-Ub^LF^)) in the presence of DNA (ФX174 RFI dsDNA, 11.1 nM) for 60 min at 25°C. Salt concentrations were as indicated. Reactions were subjected to SDS-PAGE followed by staining with InstantBlue Coomassie protein stain and western blotting.

Next, we tested whether enhancement of SPRTN autocleavage by monoubiquitylation stems from a direct effect on the enzyme's activity. To this end, we produced recombinant SPRTN-Ub^LF^ and compared its autocleavage and substrate cleavage activity to WT SPRTN. Indeed, SPRTN-Ub^LF^ displays markedly increased DNA-dependent autocatalytic cleavage *in vitro* (Figure 6A). The effect is particularly strong in the presence of dsDNA and in high salt conditions. In contrast, cleavage of histone H1 or that of model DPC substrates (fluorescently-labelled protein G-oligonucleotide conjugates) is not significantly increased (Figure 6A and B). SPRTN autocleavage occurs *in trans* with one SPRTN molecule cleaving a second (8,9). A catalytically inactive Flag-tagged SPRTN-EQ variant is cleaved more efficiently by SPRTN-Ub^LF^ than by WT SPRTN. This suggests that modification of the SPRTN molecule cleaving *in trans* is sufficient to enhance autocleavage (Figure 6C). These *in vitro* data demonstrate that enhanced autocleavage of SPRTN-Ub in cells is caused by a direct effect of the modification on SPRTN activity. Thus, we conclude that monoubiquitylation negatively controls the SPRTN pool not only by inducing proteasomal degradation *in cis* but also by triggering the inactivation of other SPRTN molecules through *in trans* autocleavage.

## DISCUSSION

**Figure 7.**
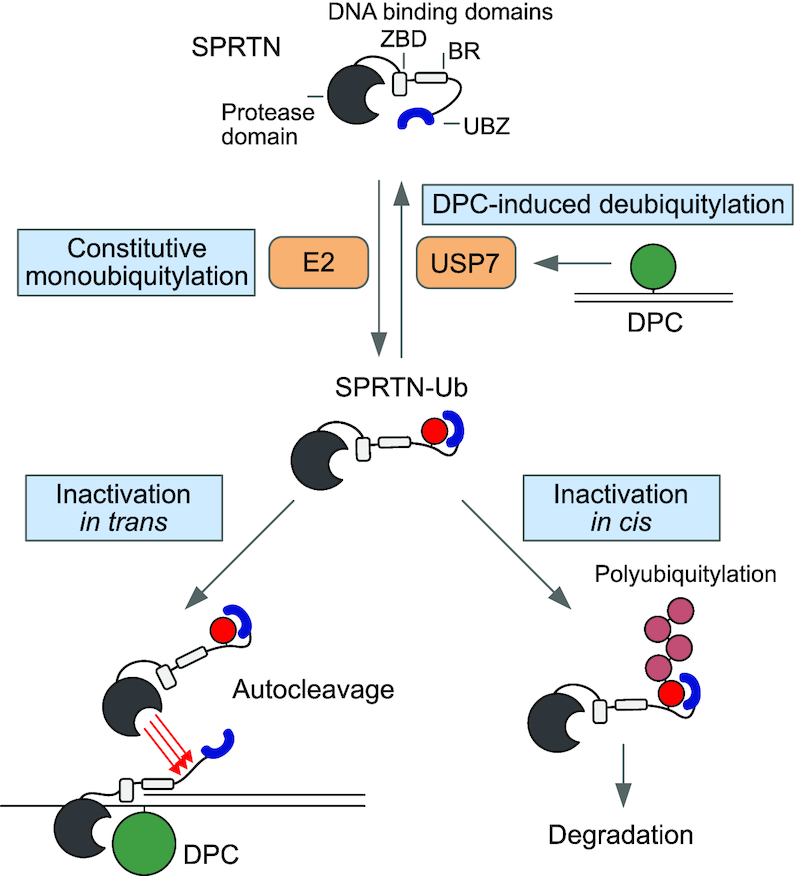
Regulation of SPRTN by monoubiquitylation and USP7. Proposed model for the regulation of SPRTN by monoubiquitylation and USP7-mediated deubiquitylation. SPRTN is subjected to constitutive promiscuous monoubiquitylation of its C-terminal tail. The modification is shielded by SPRTN’s ubiquitin binding zinc-finger (UBZ). Monoubiquitylation affects SPRTN twofold. It primes SPRTN *in cis* for proteasomal degradation by inducing polyubiquitylation while also triggering inactivation by fostering autocleavage of other SPRTN molecules *in trans*. USP7 relieves this inhibition by deubiquitylating SPRTN upon induction of DNA–protein crosslinks (DPCs).

The regulatory ubiquitin switch revealed here is distinct from other prominent types of monoubiquitylation events occurring during genome maintenance. FANCD2 and FANCI are ubiquitylated by the Fanconi anemia core complex in a site-specific manner upon recruitment to chromatin during the repair of inter-strand crosslinks (33). PCNA is site-specifically monoubiquitylated as a response to stalled DNA synthesis, which fosters recruitment of translesion synthesis polymerases (34–37). In contrast, SPRTN monoubiquitylation is not triggered by DPC induction but instead appears to be a constitutive process. Our data demonstrate that the modification can have two distinct outcomes, both of which lead to inactivation of the enzyme (Figure 7). Firstly, monoubiquitylation primes SPRTN *in cis* for proteasomal degradation by fostering polyubiquitylation. Secondly, it further reduces the amount of active SPRTN by promoting autocleavage *in trans*. Importantly, SPRTN autocleavage requires the presence of DNA *in vitro*, which infers that monoubiquitylation-triggered autocleavage in cells is specifically affecting DNA-bound SPRTN molecules. If cells face DPC induction, USP7-mediated deubiquitylation appears to be important to stall this negative regulation in order to prolong the half-life of active DNA-bound SPRTN. Formaldehyde exposure triggers wide-spread ubiquitylation events in cells (38). It is thus attractive to speculate that in the presence of DPCs SPRTN’s UBZ domain engages with specific ubiquitylation signals. In turn, this would expose the monoubiquitylation and thereby allow USP7 to remove the modification. Although the regulation described here is important to control SPRTN protein levels, it does not participate in the recruitment of SPRTN to chromatin. This finding is further supported by the fact that the RJALS syndrome variant SPRTN-ΔC retains a large degree of function despite lacking the UBZ and not being monoubiquitylated. In this context, it will be interesting to investigate whether the phenotypes observed in RJALS are caused, at least in part, by the loss of the negative regulation of SPRTN and not only by a reduction in DPC repair capacity. Notably, recruitment of SPRTN to UV-induced lesions (but not DPCs) has been shown to depend on the UBZ domain potentially indicating that this domain serves dual purposes (20–22). However, how SPRTN is recruited to DPCs remains controversial with evidence pointing towards ubiquitylation or SUMOylation signals (39,40). Understanding how the presence of crosslinks is signalled to DPC repair enzymes will be critical to decipher decision making during DPC repair. The recent identification of several additional proteases targeting DPCs implies that DPC repair pathway choice is a complex cellular process (13,39,41–45). At any rate, the intricate negative regulation described here highlights not only the complexity of DPC repair but also the importance of controlling SPRTN’s potentially toxic proteolytic activity.

## DATA AVAILABILITY

Data are available from the corresponding author upon reasonable request.

## Supplementary Material

gkaa1224_Supplemental_File
