# Supplementary material for: A ubiquitin switch controls autocatalytic inactivation of the DNA–protein crosslink repair protease SPRTN
Source: Nucleic Acids Res. 2020 Dec 22;49(2):902–15. doi: 10.1093/nar/gkaa1224 (PMC7826251; doi:10.1093/nar/gkaa1224)

SUPPLEMENTARY DATA

A ubiquitin switch controls autocatalytic inactivation of the DNA-protein crosslink repair protease SPRTN

Shubo Zhao, Anja Kieser, Hao-Yi Li, Hannah K. Reinking, Pedro Weickert, Simon Euteneuer, Denitsa Yaneva, Aleida C. Acampora, Maximilian J. Götz, Regina Feederle and Julian Stingele

Figure S1 (related to Figure 1). E3-independent monoubiquitylation SPRTN *in vitro*.

A. Analysis of monoubiquitylation of truncated SPRTN variants. Plasmids encoding the Strep-tagged SPRTN-Δ400 truncation (carrying the indicated lysine to arginine (KR) substitutions. K407 was not replaced in the SPRTN-Δ400-8KR variant) were transiently transfected in HeLa T-REx Flp-In cells. Expression of SPRTN was induced by addition of doxycycline for 16 hours before cells were lysed and subjected to immunoprecipitation using anti-Strep beads followed by western blotting. Western blotting of cell lysates against Tubulin serves as loading control.

B. Twenty-nine human E2 ubiquitin conjugating enzymes (2 µM) were incubated together with SPRTN-EQ (2 µM), E1 ubiquitin activating enzyme (100 nM), ubiquitin (50 µM), DNA (11.1 nM ΦX174 single-stranded DNA) and ATP (2 mM) for 1.5 hours at 30°C. Reactions were stopped by addition of LDS sample buffer and subjected to SDS-PAGE followed by staining with InstantBlue Coomassie protein stain.

C. *In vitro* ubiquitylation assays containing SPRTN-EQ (410 nM), E1 ubiquitin activating enzyme (300 nM), UBE2D3 (16 µM), ubiquitin WT or no-Lys, N-terminally biotinylated ubiquitin (50 µM) and ATP (2 mM) were incubated for 1.5 hours at 30°C. Reactions were stopped by addition of LDS sample buffer and subjected to SDS-PAGE followed by staining with InstantBlue Coomassie protein stain.

Figure S1 (related to Figure 1)

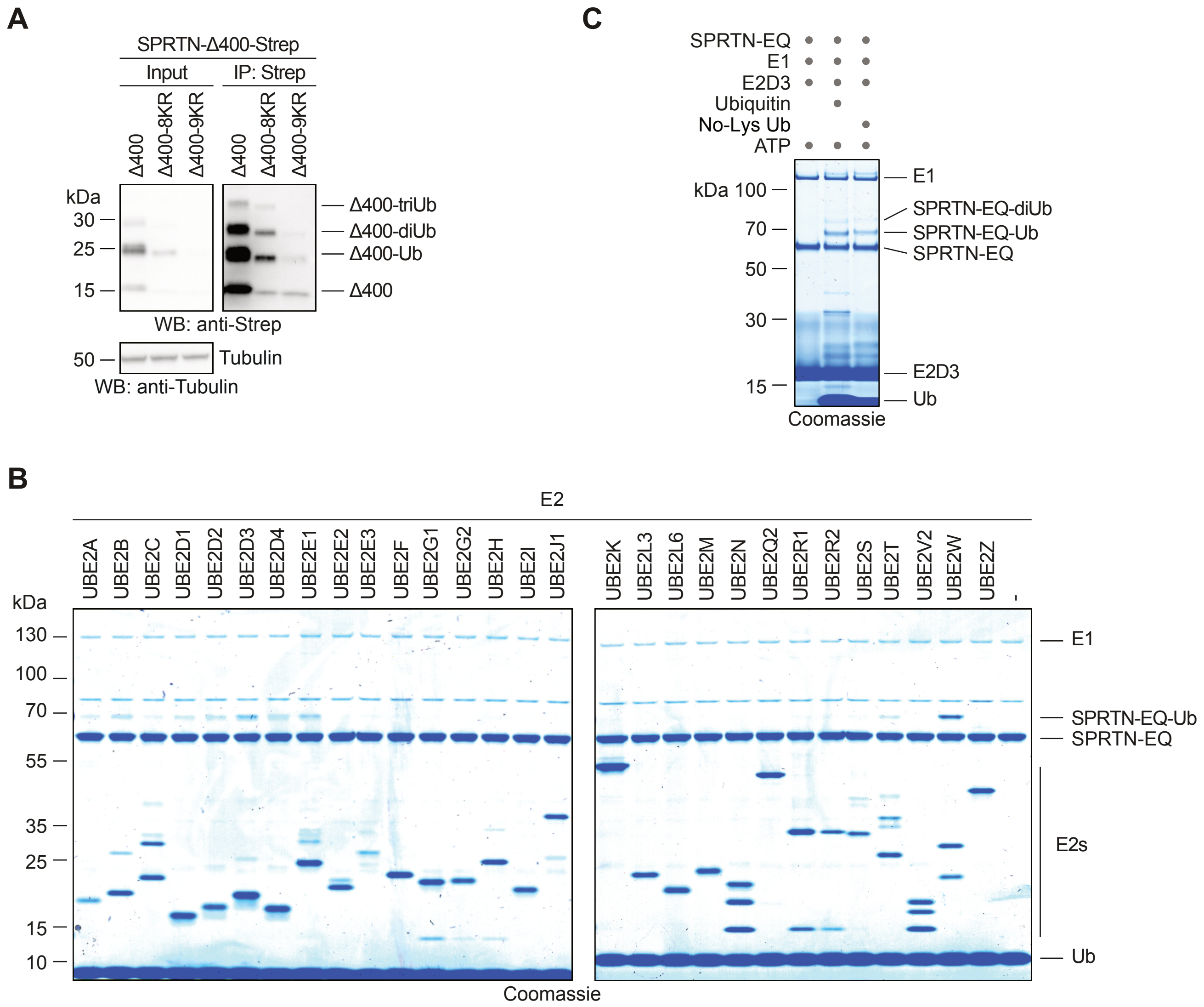

Figure S2 (related to Figure 2). An *in vitro* screen reveals that USP7 targets ubiquitylated SPRTN.

A. Result of the primary screen schematically depicted in Figure 2A. Twenty-four pools (each containing three different lysates of *E. coli* cells expressing different DUB cDNAs) were incubated for 30 min at 25°C together with purified partially-ubiquitylated YFP-SPRTN-EQ-Strep in three experimental sets (A-K, L-N, O-X). Reactions were stopped by addition of LDS sample buffer and analysed by SDS-PAGE and western blotting using anti-Strep antibody. Lysates of BL21 cells served as negative control, the unspecific deubiquitylating activity of the catalytic domain of the USP2 ($USP2^{cd}$) as positive control.

B. Deubiquitylation activity of indicated partially purified DUBs was compared using the commercial Ubiquitin-Rhodamine cleavage assay, which measures the release of a rhodamine fluorophore C-terminally conjugated to ubiquitin with cleavage resulting in increased fluorescence. Left panel, increase in rhodamine fluorescence over time. Right panel, initial velocities of the deubiquitylating reactions. The catalytic domain of the USP2 ($USP2^{cd}$) served as positive control. Values represent the mean ± SD of two technical replicates.

C. Indicated partially purified DUBs were incubated for 30 min at 25°C together with purified partially-ubiquitylated YFP-SPRTN-EQ-Strep. Reactions were stopped by addition of LDS sample buffer and analysed by SDS-PAGE and western blotting using anti-Strep antibody.

Figure S2 (related to Figure 2)

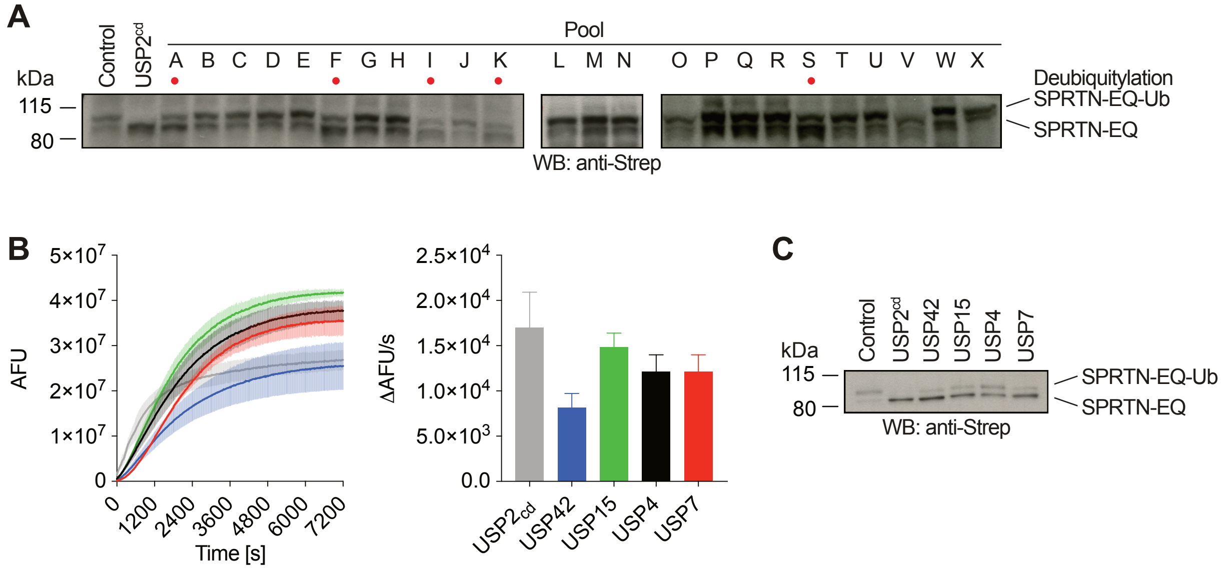

Figure S3 (related to Figure 4). USP7 deubiquitylates SPRTN upon DPC induction.

A. Western blot analysis of DLD1 WT or *USP7* KO cells transiently transfect with empty vector, YFP-tagged USP7 WT or the catalytically inactive USP7 CS variant, as indicated. Asterisks indicates a cross-reactive band.

B. Plasmids encoding Flag-tagged full-length USP7, VCPIP1 or USP11 (WT or catalytically inactive variants) or the empty vector were transiently transfected in HeLa T-REx Flp-In cells stably expressing doxycycline-inducible YFP-SPRTN-Strep. Binding was analysed by co-immunoprecipitation using anti-Flag beads followed by western blotting.

C. DLD1 cells transfected with siRNA pools targeting USP7, VCPIP1 or USP11 were treated for 2 hours with 2 mM formaldehyde (FA) 72 hours after transfection. Cells were lysed in LDS sample buffer and analysed by SDS-PAGE followed by western blotting.

D. HAP1 WT, *USP11* KO and *VCPIP1* KO cells were treated with the indicated formaldehyde concentrations for 72 hours. Cell viability was then determined using the alamarBlue cell viability assay. Values represent the mean ± SD of three technical replicates normalized to the mean of untreated controls of each cell line.

E. HAP1 WT, *USP7* KO, *VCPIP1* KO and *USP11* KO cells were treated with 2 mM formaldehyde (FA) for 3 hours. Cells were either lysed directly in LDS sample buffer (total) or subjected to chromatin fractionation. Samples were then analysed by SDS-PAGE followed by western blotting. Asterisks indicates a cross-reactive band.

Figure S3 (related to Figure 4)

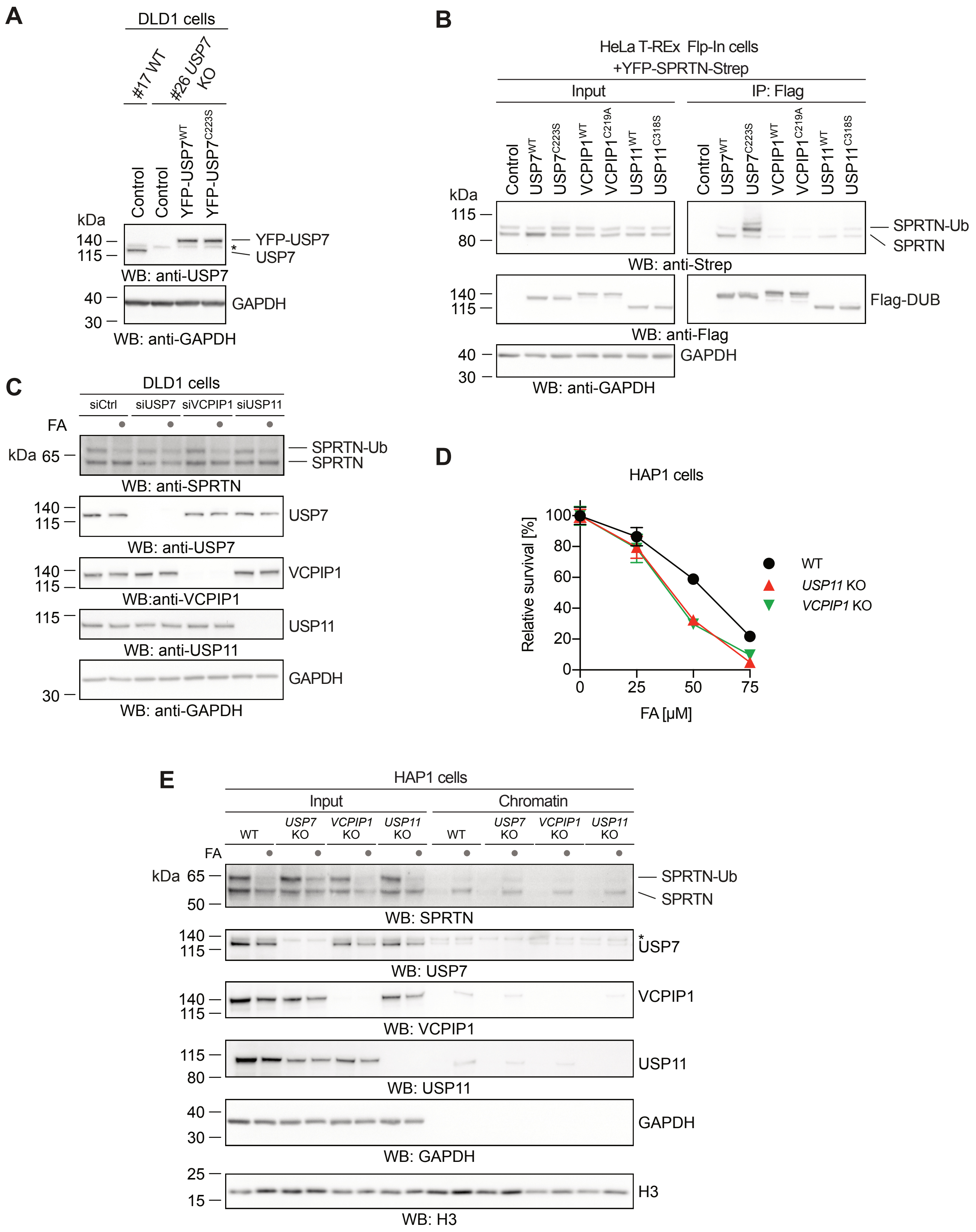

Figure S4 (related to Figure 5). Monoubiquitylation promotes SPRTN degradation and autocleavage.

A. Stability of endogenous SPRTN was determined in HCT116 WT or *USP7* KO cells using a cycloheximide-chase experiment. Cells were incubated in the presence of cycloheximide for the indicated amount of time (with or without a 2-hour pre-treatment with the proteasome inhibitor MG132) prior to cell lysis and analysis by western blotting.

B. Stability of stably expressed doxycycline-inducible YFP-SPRTN-Strep and YFP-SPRTN-UBZ*-Strep was determined in HeLa-T-REx Flp-In cells using a cycloheximide-chase experiment. Cells were incubated in the presence of cycloheximide for the indicated amount of time (with or without a 2-hour pre-treatment with the proteasome inhibitor MG132) prior to cell lysis and analysis by western blotting.

C-D. HAP1 or HCT116 cells were treated with proteasome inhibitor MG132 for the indicated amount of time prior to cell lysis and analysis by western blotting. Asterisks indicate autocleavage fragments.

E-F. Stability of stably expressed doxycycline-inducible YFP-SPRTN-Strep and catalytically inactive YFP-SPRTN-EQ-Strep or the truncated YFP-SPRTN-aa1-227-Strep was determined in HeLa-T-REx Flp-In cells using a cycloheximide-chase experiment. Cells were incubated in the presence of cycloheximide for the indicated amount of time (with or without a 2-hour pre-treatment with the proteasome inhibitor MG132) prior to cell lysis and analysis by western blotting.

Figure S4 (related to Figure 5)

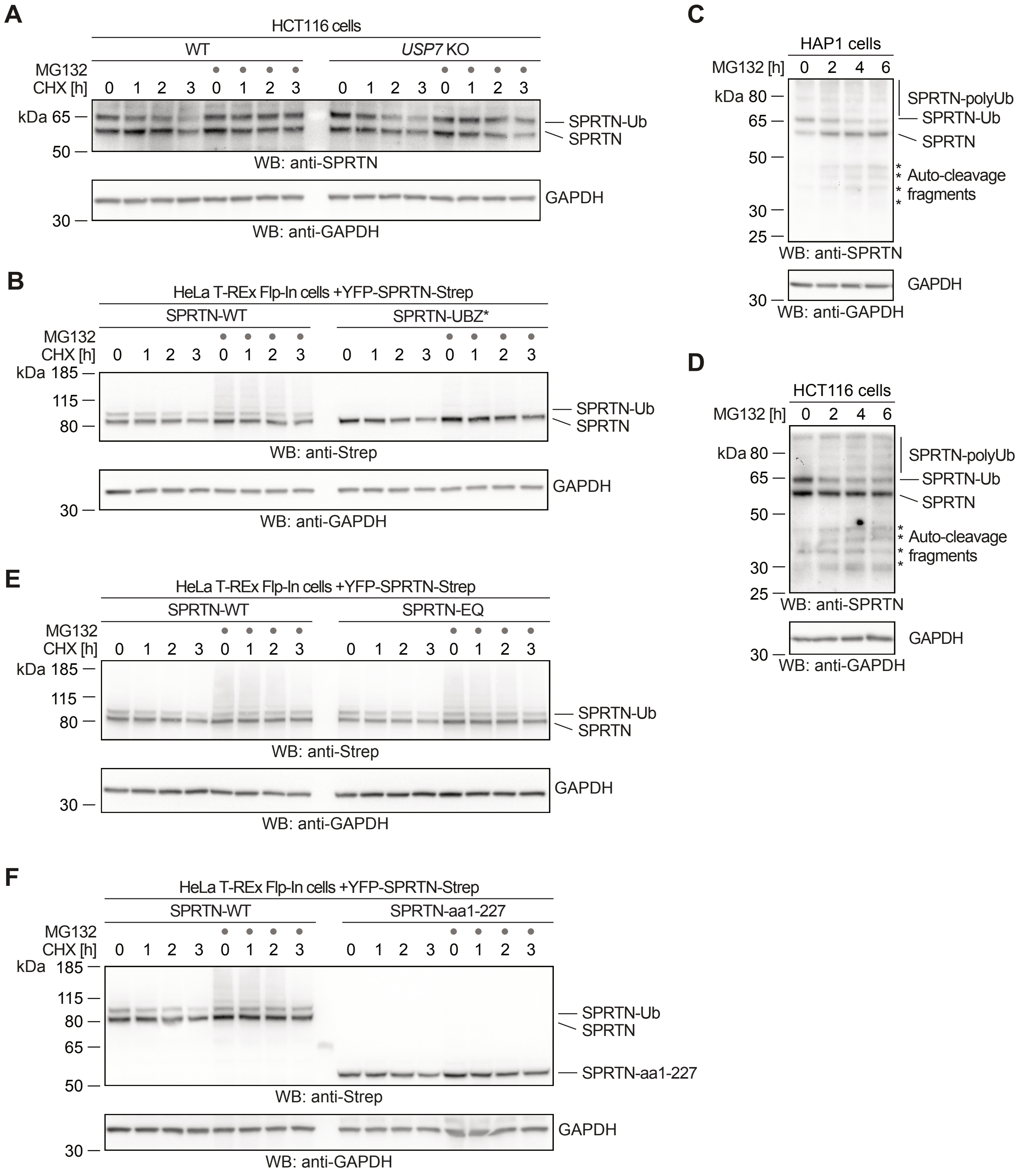

Supplement: gkaa1224_Supplemental_File [file gkaa1224_supplemental_file.pdf]
